# Age‐dependent effect between MARCO and TLR4 on PMMA particle phagocytosis by macrophages

**DOI:** 10.1111/jcmm.14494

**Published:** 2019-06-21

**Authors:** Chiaki Yamada, Camila Beron‐Pelusso, Neira Algazzaz, Alireza Heidari, Diogo Luz, Mutasem Rawas‐Qalaji, Ion Toderas, Ana Karina Mascarenhas, Toshihisa Kawai, Alexandru Movila

**Affiliations:** ^1^ College of Dental Medicine Nova Southeastern University Fort Lauderdale Florida; ^2^ Halmos College of Natural Science and Oceanography Nova Southeastern University Fort Lauderdale Florida; ^3^ College of Pharmacy Nova Southeastern University Fort Lauderdale Florida; ^4^ Institute of Zoology Academy of Sciences of Moldova Chisinau Republic of Moldova; ^5^ Institute for Neuro‐Immune Medicine Nova Southeastern University Fort Lauderdale Florida

## Abstract

Progressive generation of total joint implant‐derived wear particles is one of the major risk factors in development of peri‐prosthetic osteolysis especially in the aging society. It is commonly accepted that macrophages predominantly drive the inflammatory response to wear debris particles. Among various surface receptors that activate the macrophages to phagocytize particles, it is believed that the Toll‐like receptor 4 (TLR4) and the scavenger macrophage receptor with collagenous structure (MARCO) play key roles in recognition of wear debris particles. However, a strong body of evidence indicates an age‐dependent diminished function of human TLRs. Thus, we hypothesized that the MARCO receptor may be more engaged than TLRs in the phagocytosis of wear debris particles which in turn up‐regulate production of pro‐inflammatory cytokines from aged macrophages. We demonstrated that peritoneal macrophages isolated from aged mice show elevated expression of MARCO receptor compared to that from young mice. In contrast the expression of TLR4 was significantly decreased on the surface of aged macrophages. Furthermore, using anti‐MARCO and anti‐TLR4 neutralizing mAbs, we demonstrated the age‐dependent pathogenic role of MARCO, but not TLR4, receptor in promoting poly‐methyl methacrylate (PMMA) bone cement particles phagocytosis by macrophages leading to the release of pro‐inflammatory cytokines migration inhibitory factor and tumour necrosis factor in vitro. These data also suggest that the approach to neutralize MARCO may lead to the development of therapeutic regimen for the prevention of particle‐induced osteolysis in aged patients.

## 1 INTRODUCTION

Approximately one million arthroplasty surgeries are performed annually, whereas about 20% of patients need implant replacement within a decade from initial surgery as a result of developing particle‐induced osteolysis.^1^ It has been observed that particles generated from the prosthetic joint articular surface as well as from the artificial bone cements are the major factors determining the survival of joint implants leading to the development of bone osteolysis.^1^ Multiple lines of evidence indicate that patients aged 65+ are more susceptible to bone peripheral lesions compared to younger individuals, indicating a critical need for the development of new strategies to prevent or treat pathological bone osteolysis in senior individuals.^2^

Our group reported that particle‐stimulated macrophages (Mф) play an essential role in producing of pro‐inflammatory chemokines and cytokines, such as macrophage migration inhibitory factor (MIF) and tumour necrosis factor (TNF)‐α, in the particle‐induced bone osteolysis lesions via observation of young mice.^3^ Among various surface receptors that activate the macrophages to phagocytize particles, it is believed that the innate immune Toll‐like receptor 4 (TLR4) and the scavenger receptor macrophage receptor with collagenous structure (MARCO) play key roles in recognition of wear debris particles in the context of particle‐induced bone osteolysis.^4^ However, since a strong body of evidence supports the fact that human TLRs function is diminished in an age dependent manner,^5^, ^6^ it is plausible that the MARCO receptor may be more engaged than TLRs in the phagocytosis of wear debris particles which in turn up‐regulate inflammation in bone osteolysis of aged patients. Nevertheless, to the best of our knowledge, no study has ever addressed the role of TLR4 and MARCO receptors in the particle‐phagocytosis and production of pro‐inflammatory cytokines by Mф in relation to aging.

In the present study, we report that MARCO receptor appears to play a critical role in the in vitro phagocytosis of bone cement poly‐methyl methacrylate (PMMA) particles by peritoneal Mф (pMф) isolated from aged mice, causing excess productions of various pro‐inflammatory cytokines (MIF and TNF‐α).

## 2 MATERIAL AND METHODS

Given space constraints in the main text, detailed Material and Methods can be found in the Supplementary Material and Methods file.

## 3 RESULTS

### 3.1 Aging impairs expressions of MARCO and TLR4 receptors on the surface of pMф

**Figure 1:**
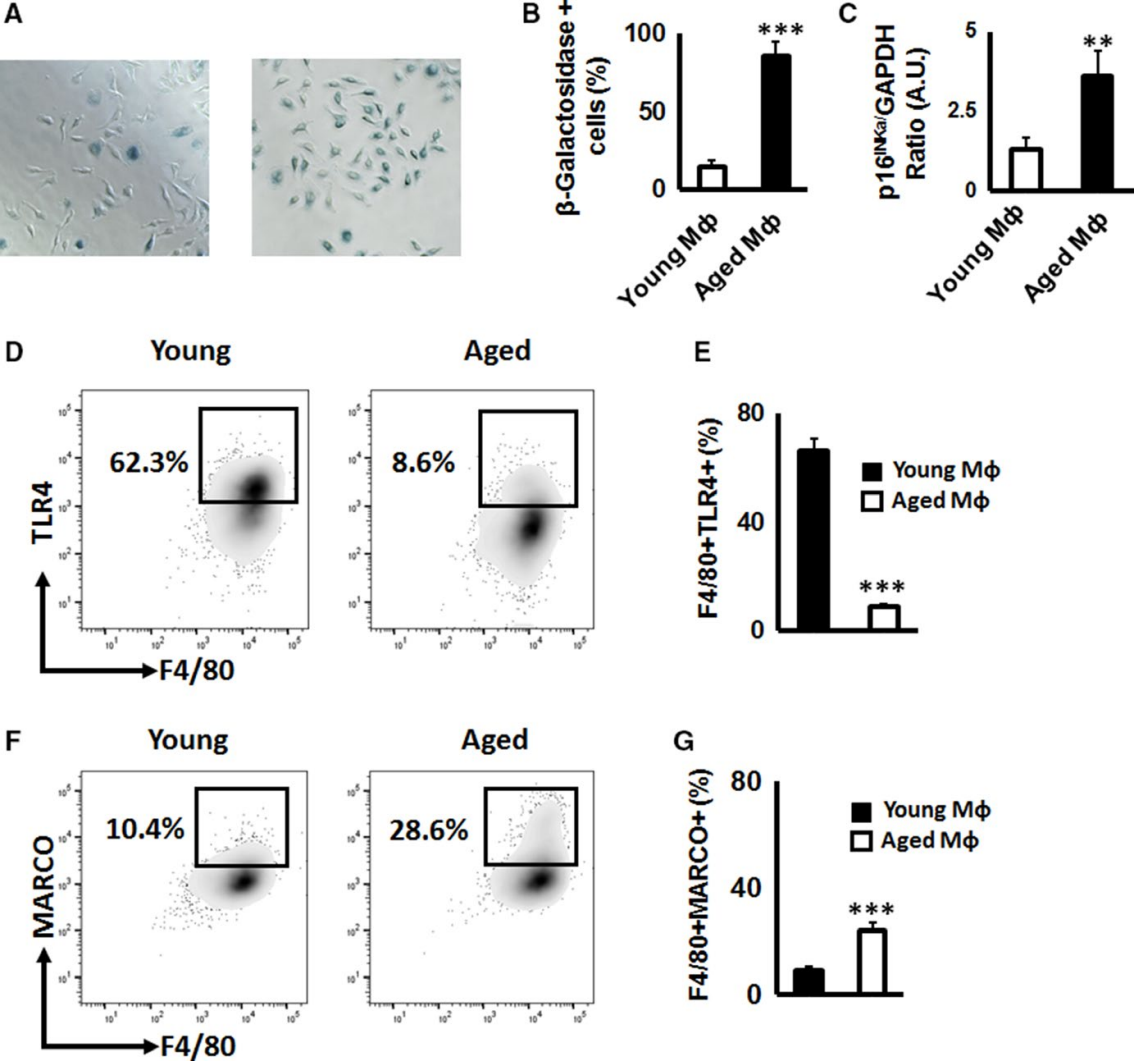
Age‐dependent expression of Toll‐like receptor 4 (TLR4) and macrophage receptor with collagenous structure (MARCO) receptors on the surface of peritoneal macrophages (pMф). Representative images (A) and quantification (B) of β‐galactosidase activity, as well as expression of p16^INKa^ mRNA (C), senescence‐associated markers, in pMф isolated from young (2‐month‐old) and aged mice (24‐month‐old animals). The number of blue β‐galactosidase positive senescent cells was quantified microscopically. (D, E) Representative contour plots and percentage of F4/80 + TLR4 + pMф isolated from young and aged mice. (F,G) Representative contour plots and percentage of F4/80 + MARCO + pMф isolated from young and aged mice. The fluorescence activated cell sorting (FACS) data are based on the 50 000 collected events. All experiments were repeated at least three times in quadruplicate/condition. **P < 0.01, ***P < 0.001

First, to confirm the cellular senescence, pMф isolated from young and aged animals were evaluated for the β‐galactosidase activity as well as for p16^INKa^ mRNA gene expression. As expected, the number of β‐galactosidase positive cells was significantly elevated in pMф isolated from aged mice than those isolated from young animals (Figure 1A,B). In addition, expression of p16^INKa^ was significantly up‐regulated in pMф isolated from aged mice compared to that of young mice (Figure 1C), indicating that pMф in aged mice exhibit a senescence‐associated phenotype.

Because it is reported that TLR4 and MARCO contribute to the cellular interaction with particles produced from the implant device,^4^ we evaluated next the expression of TLR4 and MARCO receptors on the surface of mouse F4/80 + pMф. The expression of TLR4 was significantly decreased on the surface of aged F4/80 + pMф compared to that of young F4/80 + pMф (Figure 1D,E). In contrast, the expression level of MARCO on the surface of aged F4/80 + pMф was comparable to the young pMф (Figure 1F,G). Taken together, these results suggest that aged pMф have elevated expression of MARCO, but not TLR4 receptor compared to that of young pMф. These results indicate that MARCO could be engaged in the interaction with PMMA particles by macrophages in particle‐induced lesions of elderly patients.

### 3.2 MARCO receptor is required for PMMA particles‐induced inflammation in relation to aging

Because it was reported that one of the positively charged domains of MARCO plays an essential role in binding to the negatively charged Titanium (Ti) particles by alveolar Mф,^7^ we measured first the Z‐potential of organic bone cement PMMA particles and compared it with Ti particles. The average Z‐potential of PMMA and Ti particles were −52.6 ± 1.5 and −27.9 ± 5.1 mV, respectively (Table S1), indicating that both negatively charged PMMA and Ti particles may interact with the positively charged MARCO.

**Figure 2:**
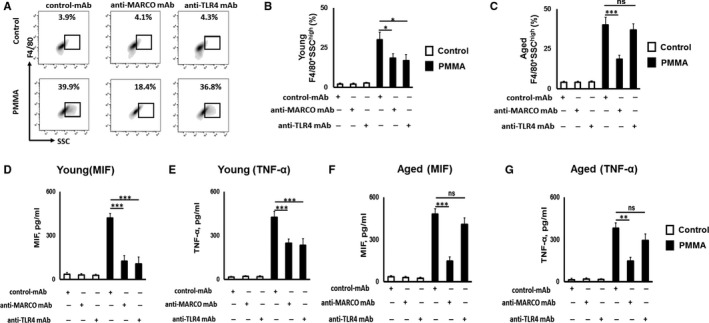
Effects of anti‐macrophage receptor with collagenous structure (MARCO) and anti‐Toll‐like receptor 4 (TLR4) mAbs on promoting poly‐methyl methacrylate (PMMA) particles phagocytosis and production of pro‐inflammatory cytokines from aged peritoneal macrophages (pMф) in vitro. Representative contour plots (A) and percentage of young F4/80^+^SSC^high^ (B) and aged F4/80^+^SSC^high^ (C) pMф exposed to PMMA particles. Altogether 50 000 fluorescent events were collected. Concentrations of migration inhibitory factor (MIF) (D, F) and tumour necrosis factor (TNF)‐α (E, G) detected from PMMA‐stimulated young and aged pMф, respectively. pMф were exposed to PMMA particle at a ratio of 1:300 in the presence or absence of control Ab, anti‐MARCO or anti‐TLR4 Abs for 24 h. No particle stimulated cells were used as a control. All experiments were repeated at least three times in quadruplicate/condition **P < 0.01, ***P < 0.001; ns, not significant

Next, to examine whether neutralization of MARCO as well as TLR4 could affect phagocytosis of PMMA particles by aged and young pMф in vitro, PMMA particles were incubated with pMф isolated from aged and young mice, in the presence of control IgG mAb, anti‐MARCO mAb and anti‐TLR4 mAb and then were analysed by multicolour flow cytometry (Figure 2A). Addition of PMMA particles significantly up‐regulated the SSC value compared to non‐particle treated F4/80 + pMф isolated from both young and aged mice (Figure 2B,C). However, the MARCO‐specific blocking Ab significantly suppressed the increase of SSC in response to stimulation with PMMA‐particles by F4/80 + pMф isolated from both young and aged mice, indicating that MARCO receptor is engaged in the internalization of PMMA particles by both young and aged pMф. In contrast, anti‐TLR4 mAb did not show any effect on PMMA particles internalization by aged pMф (Figure 2C). However, anti‐TLR4 mAb significantly suppressed the phagocytosis of PMMA‐particles by pMф isolated from young mice (Figure 2B). These results indicate that both MARCO and TLR4 receptors are engaged in particle phagocytosis by young pMфs, while MARCO become the dominant receptor in relation to diminished TLR4 in phagocytosis of PMMA particles by aged pMф.

### 3.3 Anti‐marco neutralizing mAb reduces secretion of pro‐inflammatory cytokines from PMMA particle‐stimulated pMФ

Because we reported earlier that pro‐inflammatory cytokines MIF and TNF‐α are engaged in the particle‐induced bone osteolysis,^3^ we compared the effects of anti‐MARCO and anti‐TLR4 mAbs on the production of these cytokines by aged and young pMф in response to PMMA particles. Our results indicate that both anti‐MARCO and anti‐TLR4 mAbs significantly down‐regulated productions of pro‐inflammatory cytokines, that is, MIF and TNF‐α, by young pMфs stimulated with PMMA particles (Figure 2D,E). In contrast, the anti‐MARCO mAb significantly down‐regulated production of MIF and TNF‐α from PMMA‐particle stimulated pMф that were isolated from aged mice (Figure 2F,G). No effect of anti‐TLR4 mAb on production of pro‐inflammatory cytokines by PMMA stimulated aged pMфs was detected (Figure 2F,G). Collectively, these data suggest that, in relation to aging, MARCO appears to be responsible for the productions of pro‐inflammatory cytokines, MIF and TNF‐α, by PMMA‐stimulated aged pMфs in vitro.

## 4 DISCUSSION

This study demonstrated that both young and aged Mф are able to phagocyte PMMA‐particles in a MARCO‐dependent manner, confirming the earlier published observation that MARCO expressed on Mф is one of the major binding receptors for particles.^8^ It is true that, although TLR4 and MARCO expressed on Mф were reported to be the receptors for particles in peripheral osteolytic lesions induced in young mice,^4^, ^8^ the effects of aging on these receptors' binding ability to particles in relation to the pathogenesis of particle‐induced osteolysis was not addressed by the previous studies. Here, we demonstrated that pMф isolated from aged mice show diminished expression of TLR4 on their surface compared to that of pMф isolated from young mice. These data are in line with earlier published evidence indicating the age‐dependent decline in TLR expression on the surface of Mфs.^9^ Additionally, we demonstrated that pMфs isolated from aged mice show elevated expression of MARCO receptor compared to the pMф isolated from young mice. Furthermore, our results are in accordance with the report showing the age‐dependently elevated expression of MARCO on the surface of microglia.^10^ On the other hand, it was shown that there is no difference in the level of MARCO expression between young and aged alveolar Mф.^11^ Importantly, expression of MARCO was significantly up‐regulated in lipopolysaccharide‐induced tolerant Mф.^12^ Although Mф could phagocyte wear‐debris particles through the scavenger receptor and complement receptor 3,^13^ the role of these receptors in the phagocytosis of PMMA particles by Mф in relation to aging remain elucidated.

It is clear that Mф are an important cellular source of pro‐inflammatory cytokines, including MIF and TNF‐α, and that they play a key role in the development of age‐dependent bone osteolytic lesions.^3^, ^14^ In the present study, anti‐MARCO mAb, but not anti‐TLR4 mAb, significantly suppressed productions of MIF and TNF‐α in vitro from aged pMф in response to 5.0 µm PMMA particles, corresponding to the report showing the highest production of pro‐inflammatory cytokines by bone‐marrow derived mouse macrophages in response to 1‐6 µm particle size.^15^ While comprehensive in vivo assessment of MARCO' and TLR4's roles in age‐dependent pathogenic bone resorption is required in future studies, the present study gained insight into pathophysiology of wear debris‐induced osteolysis in relation to aging.

In conclusion, we demonstrated that MARCO receptor, but not TLR4, appears to play a key pathogenic role in promoting phagocytosis of PMMA particles by aged Mф which, in turn, elicits the release of pro‐inflammatory cytokines in vitro.

## CONFLICT OF INTEREST

The authors confirm that there is no conflict of interest.

## AUTHORS CONTRIBUTIONS

A. Movila designed the experiment; C. Yamada, C. Beron‐Pelusso, N. Algazzaz, A. Heidari, D. Luz, M. Rawas‐Qalaji, I. Toderas, AK Mascarenhas, T. Kawai performed the experiments and analysed the data. C. Yamada, T. Kawai, and A. Movila wrote the manuscript.

## Data Availability

All relevant data are within the paper and its Supporting Information files.
