# Supplementary material for: Age‐dependent effect between MARCO and TLR4 on PMMA particle phagocytosis by macrophages
Source: J Cell Mol Med. 2019 Jun 21;23(8):5827–31. doi: 10.1111/jcmm.14494 (PMC6653467; doi:10.1111/jcmm.14494)
Supplement: Supplementary file 1 [file JCMM-23-5827-s001.docx]

| **Table S1:** Physical characterization of PMMA and Ti particles |
| --- |

| Particles | T (C^0^) | Particles size, µm | Z potential, mV |
| --- | --- | --- | --- |
| PMMA | 25.0 | 5.0±1.1 | -52.6±1.6 |
| Ti | 25.0 | 3.5±0.3 | -25.4±9.2 |

Poly-methyl methacrylate (PMMA; Bangs Laboratories) and Titanium (Ti; Sigma) particles were suspended in deionized distilled water (0.15% w/v). Then, the particles size distribution (Z-average: µm) and charges of particles (Zeta Potential: mV) were measured by Zetasizer ZS90 (Malvern Panalytical Ltd).
