# Supplementary material for: Age‐dependent effect between MARCO and TLR4 on PMMA particle phagocytosis by macrophages
Source: J Cell Mol Med. 2019 Jun 21;23(8):5827–31. doi: 10.1111/jcmm.14494 (PMC6653467; doi:10.1111/jcmm.14494)
Supplement: Supplementary file 2 [file JCMM-23-5827-s002.docx]

**Supplementary Material and Methods**

**Isolation of peritoneal macrophages (pМф) and evaluation of their expressions in markers associated with cellular senescence**

Resident pМфs were isolated by lavage of the peritoneal cavity from both young (two-month-old) and aged (twenty-four-month-old) wild type C57BL/6 mice. Collected adherent pМф were evaluated for expression of p16^INKa^ mRNA, β-galacatosidase activity and the senescent-associated marker, using Real-Time PCR and β-Galactosidase Staining Kit (Cell Signaling Technology) as described [1].

**PMMA particle stimulation of pМф**

pМф were stimulated with PMMA particles (diameter 5.0 µm) as described earlier [2]. Briefly, 1 x 10^5^ pМф were incubated with a suspension of PMMA particles at a ratio of 1 : 300 (cell : particle) in the presence or absence of anti-MARCO (R&D), anti-TLR4 (BioLegend) mAbs, or IgG control at a concentration of 50 μg/ml. No particle-containing medium served as a control.  After 24 h of stimulation, pМф were analyzed by an EVOS cell imaging system and multicolor flow cytometry. In addition, the culture supernatant was collected and evaluated for pro-inflammatory cytokines Macrophage Migration Inhibitory Factor (MIF) and TNF-α by ELISA. All experiments were repeated at least three times in quadruplicate/condition.

**Assay of PMMA particle phagocytosis by pМф**

Phagocytosis of PMMA particles by F4/80+ pМфs was measured by monitoring the increase of Side Scatter value (SSC^high^) using flow cytometry assay as described [3]. The increased level of SSC (granularity) from baseline (control no-stimulation) indicates the PMMA particles phagocytized by pМфs. The expressions of MARCO and TLR4 receptors on the surface of young (β-Galactosidase negative) and aged (β-Galactosidase positive) F4/80+ macrophages were also analyzed. To evaluate the multicolor fluorescetometry data, 50 000 fluorescent events were collected for respective sample and analyzed by FlowJo software.

**Quantification of β-Galactosidase positive senescent cells**

After cytochemical staining for β-galacatosidase activity, staining-patterns of cells in the culture well were acquired by a 4× objective lens (total 40x) using an EVOS cell imaging system under bright ﬁeld illumination. Then, the percentage of β-galactosidase positive senescent cells was determined by counting the number of cells showing blue staining and the total cell number in the same ﬁeld.

**Statistical analysis**

Statistical significance was evaluated using a one-way ANOVA with post hoc Tukey’s test. A p < 0.05 to be considered statistically significant.

**Supplementary References**

1. Abdul-Aziz, A.M., et al., *Acute myeloid leukemia induces protumoral p16INK4a-driven senescence in the bone marrow microenvironment.* Blood, 2019. **133**(5): p. 446-456.

2. Xing, Z., et al., *Titanium particles that have undergone phagocytosis by macrophages lose the ability to activate other macrophages.* J Biomed Mater Res B Appl Biomater, 2008. **85**(1): p. 37-41.

3. Zucker, R.M., et al., *Detection of TiO2 nanoparticles in cells by flow cytometry.* Cytometry. Part A : the journal of the International Society for Analytical Cytology, 2010. **77**(7): p. 677-685.
